# Invasive Neonatal Enterovirus Infection: A Case Series and Review of the Literature

**DOI:** 10.3390/children13091246

**Published:** 2026-09-15

**Authors:** Nicoletta Menzella, Simonetta Costa, Simona Fattore, Davide De Tomaso, Francesca Paola Fusco, Linda Sessa, Chiara Tirone, Francesca Priolo, Angela Paladini, Francesca Serrao, Tommaso Verdolotti, Rosaria Santangelo, Giovanni Vento

**Affiliations:** 1 Division of Neonatology, Department of Women and Child Health, Fondazione Policlinico Universitario A. Gemelli IRCCS, 00168 Rome, Italygiovanni.vento@unicatt.it (G.V.); 2 Ospedale Policlinico Casilino, 00169 Rome, Italy; scosta@policlinicocasilino.it (S.C.);; 3 Department of Women and Child Health Sciences, Child Health Area, Università Cattolica del Sacro Cuore, 00168 Rome, Italy

**Keywords:** enterovirus, neonatal infection, meningitis, meningoencephalitis, myocarditis, intravenous immunoglobulin, RT-PCR, neonatal sepsis

## Abstract

**Highlights:**

**What are the main findings?**

• Nine neonates with invasive enterovirus infection presented predominantly with meningitis; one developed concurrent myocarditis. All infants received IVIG and had favorable outcomes at discharge.
• Symptom onset ranged from the second to the thirty-fourth day of life; eight of nine infants developed symptoms after 72 h, a pattern compatible with either intrapartum-acquired infection (given the typical enteroviral incubation period) or postnatal acquisition, which this case series cannot distinguish with certainty.

**What are the implications of the main findings?**

• A high clinical index of suspicion for NEVI is essential, particularly when maternal or familial viral symptoms are reported around delivery, given the clinical overlap with bacterial sepsis.
• Early molecular diagnosis (RT-PCR) allows timely diagnosis and initiation of supportive management, including IVIG; however, IVIG efficacy in NEVI has not been established in controlled studies and warrants evaluation through randomized trials.

**Abstract:**

**Background/Objectives**: Enteroviruses (EV) cause neonatal infections with variable incidence and severity, occasionally progressing to invasive disease. This study reports the experience of two Italian neonatal centers with invasive neonatal EV infection (NEVI), describing management and outcomes in relation to the existing literature. **Methods:** This retrospective study included neonates diagnosed with NEVI. Data related to clinical symptoms, treatment and outcomes were reported and compared to those available in the current literature. **Results:** All nine neonates presented with systemic infection symptoms; onset ranged from the second to the thirty-fourth day of life, with eight of nine infants presenting after 72 h, most following readmission after initial discharge rather than continuous hospitalization from birth. The transmission route (vertical versus horizontal) could not be determined with certainty. All nine neonates had central nervous system infection confirmed by RT-PCR detection of EV in cerebrospinal fluid; two presented seizures or epileptiform abnormalities, and one developed myocarditis. IVIG was administered upon diagnosis. All infants survived to discharge in stable condition; neuroimaging abnormalities were documented in two infants, and long-term neurodevelopmental outcome could not be assessed in most patients due to incomplete follow-up. **Conclusions:** Invasive NEVI cannot be clinically differentiated from severe bacterial infection, warranting a high index of suspicion, particularly when caregivers report symptoms of viral infection. Further studies are needed to clarify aspects of management, including the potential benefit of IVIG administration.

## 1. Introduction

Enteroviruses (EVs) are a group of RNA viruses belonging to the Picornaviridae family and represent a significant cause of neonatal infections [1]. Neonatal EV infections (NEVIs) can range widely in their clinical presentation, from asymptomatic or mild forms to severe and life-threatening conditions [1]. The reported incidence of NEVIs varies considerably across studies and populations, highlighting their relevance to the neonatal population [2].

Of particular concern is the association of EV with neonatal meningitis, which occurs with a frequency of approximately 0.79‰ live births [3]. Beyond meningitis, the clinical spectrum of NEVI includes myocarditis, hepatitis, and systemic inflammatory responses, which can progress to septic shock and multiorgan failure [4].

On 5 May 2023, France reported an increase in cases of severe neonatal sepsis associated with Echovirus-11 (E-11) [5], a strain of a subgroup of EV. Since then, other European countries have reported cases of E-11 infection among neonates to the World Health Organization (WHO) [5]. After these events, WHO assessed the public health risk for the general population as low but encouraged countries to monitor and report cases of NEVI. The WHO also urged healthcare facilities caring for neonates to familiarize themselves with the signs and symptoms of NEVI [5].

The potential severity of clinical manifestations underscores the diagnostic challenges associated with NEVI. A recent systematic review has highlighted the global burden of perinatal and NEVI, describing a broad clinical spectrum ranging from mild disease to severe, life-threatening conditions [6]. Several risk factors have been identified for severe NEVI [7]. These include prematurity, as the underdeveloped immune systems of preterm infants limit their ability to combat viral pathogens effectively. Perinatal maternal infection also plays a critical role, as it can lead to vertical transmission of the virus, particularly during delivery [8]. Additionally, infections that manifest within the first week of life are often more severe [3], likely due to the limited transfer of maternal antibodies during the antenatal period and to the immaturity of the neonatal immune response [3]. Given these factors, early recognition and prompt management of NEVI are crucial to improving outcomes [3].

While invasive NEVIs are less common than bacterial infections, their clinical presentation is often indistinguishable, necessitating a high index of suspicion, particularly when maternal or familial viral symptoms are reported around the time of delivery. The absence of specific antiviral therapies for NEVI further complicates management, emphasizing the importance of supportive care and, in some cases, adjunctive therapies such as intravenous immunoglobulin (IVIG) to mitigate severe complications [1].

The aim of the study was to report the experience of two Italian neonatal centers with NEVI, describing the management and outcomes of neonates. The reported case series is examined in the context of existing literature.

## 2. Materials and Methods

### 2.1. Study Design, Setting and Patients

This was a retrospective observational study carried out in the neonatal intensive care units of two Italian hospitals in Rome: Fondazione Policlinico Universitario Agostino Gemelli IRCCS and Ospedale Policlinico Casilino.

Neonates admitted during a 29-month period, from May 2023 to October 2025, for symptoms suggestive of systemic infection were included if they had central nervous system infection confirmed by a positive reverse-transcription polymerase chain reaction (RT-PCR) result for EV in cerebrospinal fluid (CSF); this molecular confirmation was used as the operational definition of invasive NEVI throughout this study, encompassing both isolated meningitis and meningoencephalitis/encephalitis presentations, which are distinguished where clinically relevant. RT-PCR testing was performed by the clinical microbiology/virology laboratory of each participating center using laboratory-developed, validated molecular protocols. Further methodological detail (specific molecular target, interpretation thresholds, the approximate period during which each assay was in routine use, and whether enterovirus and parechovirus were tested as distinct targets) was not centrally available for retrospective reporting. The assays were therefore locally validated but not harmonized between centers, and cross-center differences in analytical performance could not be assessed; this is acknowledged as a limitation. Positive results were not routinely confirmed by a second assay or subjected to viral typing or sequencing.

For each neonate, we examined details about family infectious history, gestational age (GA), birth weight (BW), clinical manifestations, diagnostic test results, and treatment approaches. Clinical follow-up was also considered.

The study was conducted in accordance with institutional standards. Given the retrospective, observational design, formal Institutional Review Board approval was not required; parents or legal guardians of all included infants provided informed consent for the collection and use of clinical data.

### 2.2. Search Strategy for the Review of the Literature

This section presents a narrative (non-systematic) review of the literature on NEVIs and their management, intended to provide context for the case series rather than an exhaustive or reproducible evidence synthesis. A literature search in the MEDLINE database (via PubMed) was performed up to 31 December 2025. The following keywords were searched as entry terms: (“Enterovirus infection” OR “Coxsackievirus infections” OR “Echovirus infections”) AND (“neonate” OR “infant” OR “newborn”). All retrieved articles were screened by reading the abstract; if the abstract mentioned the NEVI, we assessed the full text for inclusion. References in the relevant papers were also reviewed, and further articles were added if necessary. Papers written in languages other than English were excluded. Available information was collected descriptively; no formal risk-of-bias or quality appraisal was performed.

## 3. Results

### 3.1. Case Presentation

Nine neonates, seven full-term and two preterm, presented with systemic symptoms of infection and were diagnosed with central nervous system infection through molecular detection of EV genome in CSF. Seven infants (Patients 1–7) presented with isolated meningitis without evidence of extraneural organ involvement; one (Patient 2) additionally developed myocarditis; and two preterm twins (Patients 8–9) presented with meningoencephalitis, with neuroimaging evidence of white matter injury, as detailed below. No patient had hepatitis or shock. Key clinical characteristics are summarized in Table 1.

#### 3.1.1. Patient 1

The first patient was a 3540 g male infant, born at 39+1 weeks of gestation with vaginal delivery. The maternal history included intrapartum fever. Neonatal symptoms began on the seventh day of life (DOL), when he was readmitted after a previous discharge, presenting with fever, irritability, and feeding difficulties. The neonate was diagnosed with meningitis, confirmed by positive EV molecular testing in the cerebrospinal fluid (CSF). Bacterial cultures were negative. Serial brain ultrasound and brain magnetic resonance imaging (MRI), at 14 DOL, showed normal findings. The patient was treated with antibiotics and IVIG at 0.5 g/kg for four days and was discharged in good clinical condition.

#### 3.1.2. Patient 2

The second patient was a 3655 g male neonate born at 37+6 weeks of gestation with vaginal delivery. Maternal and family history reported gastrointestinal symptoms before delivery. Neonatal symptoms began on the second DOL and included hypo-reactivity, pallor, capillary refill >2 s, and apnea. The neonate was diagnosed with meningitis and myocarditis, with findings of cardiac dyskinesia and mild pericardial effusion on echocardiogram, and elevated proBNP levels (2442 pg/mL at presentation, peaking at 3110 pg/mL two days later). Positive EV molecular testing in the CSF confirmed the diagnosis, whereas the bacterial cultures were negative. Treatment included antibiotics, IVIG (0.5 g/kg for four days), and dobutamine. Serial brain ultrasound and brain MRI at 14 DOL were normal. The patient was discharged in good clinical condition.

#### 3.1.3. Patient 3

The third patient was a 3835 g male neonate born at 41+0 weeks of gestation with vaginal delivery. Maternal and family history was not significant. Neonatal symptoms began on the fifth DOL, including fever, hypo-reactivity, poor feeding, and seizures. The electroencephalogram (EEG) showed normal background activity without paroxysmal abnormalities, with no indication for continuous anticonvulsant therapy. Meningitis was diagnosed, confirmed by positive EV molecular testing in CSF. Bacterial cultures were negative. Serial brain ultrasounds and brain MRI at 14 DOL were normal. The patient was treated with antibiotics, IVIG (0.5 g/kg for four days), and phenobarbital for seizure management. The neonate was discharged in good clinical condition.

#### 3.1.4. Patient 4

The fourth patient was a 2290 g female infant, born at 38+0 weeks of gestation by elective caesarean section (C-section) after regular pregnancy. Maternal history was silent for significant clinical diseases. At 13 DOL, the baby was hospitalized for fever and irritability without other associated symptoms. Meningitis was diagnosed based on positive CSF molecular testing for EV. Blood tests were normal and bacterial cultures were negative. The patient was treated with antibiotics, IVIG (0.25 g/kg for three days). Brain ultrasound was normal. Brain MRI performed three weeks after the event was normal. Neurological evaluation was normal. EEG showed good cerebral electrical activity. Cardiac evaluation showed minimal pericardial detachment without hemodynamic significance. The neonate was discharged in good clinical condition.

#### 3.1.5. Patient 5

The fifth patient was a 3960 g male infant, born at 40+3 weeks of gestation by vaginal delivery after regular pregnancy. Maternal and family history was silent for significant clinical diseases. At five DOL, the baby was hospitalized for fever associated with hypo-reactivity and poor feeding. Meningitis was diagnosed based on positive CSF molecular testing for EV. The nasal swab was also positive for EV. Bacterial cultures were negative. The patient was treated with antibiotics, IVIG (0.25 g/kg for three days). Serial brain ultrasound and brain MRI, performed five weeks after the event, were normal. Neurological evaluation showed abnormal motor activity. EEG showed good cerebral electrical activity. Cardiac evaluation showed minimal pericardial detachment without hemodynamic significance. The neonate was discharged in good clinical condition.

#### 3.1.6. Patient 6

The sixth patient was a 2550 g female infant, born at 40+2 weeks of gestation by vaginal delivery after regular pregnancy. Maternal history was silent for significant clinical diseases. At nine DOL, the baby was hospitalized for fever without other associated symptoms. Contact with an older brother with fever and viral rash was reported. Meningitis was diagnosed based on positive CSF molecular testing for EV. The nasal swab was also positive for EV. Bacterial cultures were negative. The patient was treated with antibiotics, IVIG (0.25 g/kg for three days). Brain ultrasound resulted in mild periventricular hyper-echogenicity. Brain MRI, performed two weeks after the event, was normal. Neurological evaluation showed abnormal motor activity and immaturity of neurovisual functions. EEG showed immature cerebral electrical activity. Cardiac evaluation was normal. The neonate was discharged in good clinical condition.

#### 3.1.7. Patient 7

The seventh patient was a 3450 g female infant, born at 38+1 weeks of gestation by elective C-section. Maternal history was silent for significant clinical diseases. At 30 DOL, the baby was hospitalized for fever associated with irritability and diarrhea. Meningitis was diagnosed based on positive CSF molecular testing for EV. The nasal swab was also positive for EV. Bacterial cultures were negative. The patient was treated with antibiotics, IVIG (0.25 g/kg for three days). Brain ultrasound was normal. Brain MRI performed five weeks after the event was normal. Neurological evaluation was normal. EEG showed good cerebral electrical activity. Cardiac evaluation showed minimal pericardial detachment without hemodynamic significance. The neonate was discharged in good clinical condition.

#### 3.1.8. Patient 8

Patient 8 was a small-for-GA male preterm neonate, the first-born of a monochorionic diamniotic twin pregnancy, delivered by elective cesarean section at 32+0 weeks of gestation due to selective intrauterine growth restriction. BW was 1160 g. Mild respiratory depression at birth required brief positive pressure ventilation followed by continuous positive airway pressure without oxygen supplementation. During hospitalization, a deterioration of the general clinical condition occurred, with worsening respiratory dynamics, recurrent episodes of apnea and bradycardia, and cutaneous mottling. Cerebrospinal fluid analysis and nasal swab were positive for EV, confirming the diagnosis of enteroviral encephalitis; bacterial cultures were negative. IVIG therapy was administered for four consecutive days. Serial cranial ultrasounds showed persistent bilateral periventricular hyperechogenicity. Brain magnetic resonance imaging, performed at approximately 38 weeks postmenstrual age, demonstrated diffuse deep white matter T2 hyperintensity with multiple areas of altered signal in the corona radiata and peritrigonal white matter bilaterally, considered possibly infection-associated but non-specific, with prematurity, growth restriction, and perinatal clinical instability as plausible competing contributors, together with cytotoxic–excitotoxic edema of the splenium of the corpus callosum, considered potentially reversible; no diffusion restriction, cortical or neuronal migration anomalies, or hemosiderin deposits were present, and no prior hemorrhage was identified. Electroencephalography showed age-appropriate background activity without epileptiform discharges. Cardiac evaluation documented a patent foramen ovale without hemodynamic significance. The patient was discharged in good clinical condition, with scheduled neurological and cardiological follow-up.

#### 3.1.9. Patient 9

Patient 9 was a male preterm neonate, the second-born of a monochorionic diamniotic twin pregnancy, delivered by elective cesarean section at 32+0 weeks of gestation due to selective intrauterine growth restriction of the co-twin. Birth weight was 1360 g. Mild respiratory distress at birth required non-invasive respiratory support with continuous positive airway pressure without oxygen supplementation. During hospitalization, the clinical course was complicated by worsening respiratory dynamics, recurrent episodes of apnea and bradycardia, and cutaneous mottling. Cerebrospinal fluid analysis and nasal swab was positive for EV, confirming the diagnosis of enteroviral encephalitis; bacterial cultures were negative. IVIG therapy was administered for four consecutive days. Serial cranial ultrasounds showed persistent bilateral periventricular hyperechogenicity. Brain magnetic resonance imaging, performed at approximately 38 weeks postmenstrual age, demonstrated multiple areas of altered signal (T2—hypointense; T1—hyperintense) in the centrum semiovale, corona radiata, and periventricular white matter, with linear periventricular radial hypointensities on susceptibility-weighted imaging considered compatible with deep medullary vein congestion; diffusion-weighted imaging excluded acute parenchymal injury, no hemosiderin deposits or prior hemorrhage were identified, and there was no hydrocephalus. Electroencephalography revealed a focal subclinical epileptiform abnormality over the left posterior regions, for which phenobarbital therapy (1.5 mg/kg twice daily) was initiated; no clinically evident seizures were observed. Cardiac evaluation documented a patent foramen ovale without hemodynamic significance and preserved ventricular function. The patient was discharged in good clinical condition with scheduled neurological, ophthalmological, and cardiological follow-up.

## 4. Follow-Up at One Year

One-year follow-up information is available for only three of nine patients (33%). Notably, the two preterm twins (Patients 8–9), who had the most extensive neuroimaging abnormalities in this series, had not yet reached the one-year follow-up timepoint at the time of writing; their neurological, ophthalmological, and cardiological follow-up remains ongoing as scheduled.

Patient 1 had relocated to another city and continued clinical surveillance at another center; when contacted by phone, his parents reported good health and normal development.

Patient 2 was in good condition and remained under neurological follow-up for a left intraventricular cyst first identified at brain ultrasound. A follow-up brain MRI, performed at one year of age to re-evaluate a previously known choroid plexus cyst, showed a modest increase in the size of the left intraventricular cyst, with mild ventricular ectasia and no evidence of hydrocephalus, without brain lesions related to enteroviral meningitis. Cardiac evaluation with echocardiogram was normal.

Patient 3 showed normal growth and neurological development, with no seizure recurrence and normal neuroimaging.

### Results of the Review of the Literature

Table 2 summarizes the existing literature on NEVI. The table mixes narrative reviews, cohort studies, and single case reports with non-comparable populations, which should be considered when interpreting the summarized findings.

The reviewed studies emphasize the critical importance of accurate diagnostic and treatment strategies for NEVI. Molecular diagnostics, particularly reverse transcription polymerase chain reaction (RT-PCR) testing, is fundamental in differentiating NEVI from bacterial sepsis, enabling timely interventions.

Studies highlight the role of maternal history and transmission routes, both vertical and horizontal, in guiding clinical suspicion and sample collection. Epidemiological findings underscore the seasonal nature of NEVI, with outbreaks commonly occurring in summer and fall.

Severe complications such as myocarditis, meningoencephalitis, and hepatic coagulopathy often necessitate extensive diagnostic and supportive care, including neuroimaging and laboratory evaluations. IVIG is widely reported in the literature, having been used in an estimated 41% of retrieved cases in one systematic review, although its efficacy has not been established in controlled trials [4].

## 5. Discussion

NEVIs represent a significant clinical challenge due to their wide-ranging presentations, from mild febrile illnesses to severe systemic manifestations such as myocarditis, hepatitis with coagulopathy, meningoencephalitis, and sepsis-like syndromes [4]. This clinical spectrum has been confirmed in large retrospective case series distinguishing mild and severe NEVIs [3]. These infections, as highlighted by the WHO [5], are globally prevalent and often asymptomatic but can lead to severe complications [4], mainly in the neonatal population, including hepatocellular and renal failure during outbreaks. Clinicians must maintain a high index of suspicion for NEVIs presenting with sepsis or circulatory shock and utilize molecular diagnostics, particularly RT-PCR, to confirm the diagnosis [10]. Non-enteroviral pathogens, such as norovirus, should also be considered in the differential diagnosis of neonatal seizures with white matter injury when enterovirus and parechovirus CSF testing is negative; of note, norovirus detection may require stool PCR, as CSF testing can remain negative even when norovirus is the causative agent [13]. Human parechovirus is a further important differential diagnosis, given its clinical overlap with enterovirus infection and shared classification within the Picornaviridae family; a favorable outcome following parechovirus type 5 CNS infection in a neonate has been reported [14]. Notably, enterovirus RNA may remain undetectable in the CSF despite confirmed CNS involvement, as illustrated by a case of EV-71 encephalitis with disseminated cortical and subcortical lesions on MRI and CSF pleocytosis on repeat testing, in which the virus was identified only in blood and stool [15]. Using a classification framework analogous to that proposed by Harik and DeBiasi [1], our cohort was predominantly composed of infants with isolated meningitis without extraneural organ involvement (Patients 1–7), with a minority presenting more severe phenotypes: there was myocarditis in one infant (Patient 2) and meningoencephalitis with white matter injury in two preterm twins (Patients 8–9). No patient in this series met the criteria for septic shock, hepatic necrosis, or multiorgan failure.

Regarding the mode of viral transmission, our case series showed a range of symptom onset from the second to the thirty-fourth day of life, with eight of nine infants presenting after 72 h of life. However, the enterovirus incubation period is typically 3 to 6 days, meaning infections acquired intrapartum can also manifest beyond 72 h; a >72-h onset threshold therefore cannot reliably distinguish vertical from horizontal acquisition [8]. No maternal or contact virology, serotyping, or sequencing was performed in this series, and no formal epidemiological investigation (contact tracing, cohorting, or outbreak assessment) was undertaken. We therefore report onset timing and exposure history descriptively rather than assigning a specific transmission route to individual cases. Both vertical transmission—such as intrapartum exposure to maternal vaginal secretions or, less commonly, transplacental mechanisms [8]—and horizontal transmission from symptomatic close contacts [1] remain plausible explanations consistent with the exposure histories reported in our cohort.

The distribution of maternal and household exposure histories in our cohort is consistent with the coexistence of vertical and horizontal transmission routes reported in previous NEVI series, although individual transmission events could not be confirmed virologically.

Patient 2, who presented symptoms on the second day of life, developed the most severe clinical form, with both meningitis and myocardial involvement. It has been reported that severe disease is often linked to early-onset infections within the first week of life, due to the absence of maternal serotype-specific antibodies [10]. Other risk factors for illness severity included the virulent serotypes like Coxsackievirus B or Echovirus 11 or EV 71, the latter being correlated with a high mortality rate in East Asia [16].

Similar cases of myocarditis following neonatal EV meningitis have been reported, including the case described by Bae et al., where severe cardiac dysfunction developed despite IVIG treatment [17].

In our small case series, a high index of clinical suspicion and prompt RT-PCR testing allowed timely diagnosis in all nine infants. RT-PCR testing of cerebrospinal fluid, blood, and stool has greatly enhanced our ability to identify EV early. This was important because the clinical overlap of EV infections with bacterial sepsis complicates timely diagnosis [4]. Symptoms such as fever, lethargy, respiratory distress, and poor feeding are non-specific, necessitating advanced molecular diagnostics [1]. Neuroimaging, particularly MRI, also played a crucial role in severe cases, revealing characteristic findings such as disseminated cortical and subcortical lesions that indicate central nervous system involvement [15], with imaging abnormalities correlated with long-term neurological outcomes in some series [12]. Similar case series from other geographic settings have further illustrated the varied clinical spectrum of NEVI [9,11].

It should be noted that neurological symptoms may appear. This can be explained by transient functional disturbances without structural lesions. This is what happened to our Patient 3, who presented with seizures despite normal neuroimaging [2].

In our cohort, IVIG was administered to all nine infants following NEVI diagnosis [1]; because no untreated comparator group was available, a causal effect of IVIG on the favorable outcomes observed cannot be established, and confounding by indication cannot be excluded. All infants received intravenous immunoglobulin (Kiovig^®^, human normal immunoglobulin 100 mg/mL; Takeda), although the dose and duration varied according to center-specific protocols: infants treated at Ospedale Policlinico Casilino (Patients 4–7) received 0.25 g/kg for three days, while infants treated at Fondazione Policlinico Universitario Agostino Gemelli IRCCS (Patients 1–3, 8–9) received 0.5 g/kg for four days; the rationale for each center’s protocol was not documented. In the literature, early IVIG administration has been proposed as beneficial in mitigating severe disease outcomes, particularly in cases of myocarditis and meningoencephalitis [3]; however, evidence supporting IVIG use in NEVI is derived mainly from case series and observational studies, with no definitive data from randomized clinical trials to date. Despite supportive care, severe NEVI remains associated with high mortality, with myocarditis identified as a leading driver of fatal outcomes in the largest available series [4], and survivors often face long-term neurological sequelae [4].

The literature has proposed several preventive measures for reducing NEVI incidence, including stringent infection control practices in neonatal intensive care units and targeted maternal screening during the perinatal period [1,6], as well as maternal vaccination or prophylactic IVIG in high-risk pregnancies as a potential area for future research [1]; these remain proposals from prior literature rather than conclusions supported by the present case series. Additionally, the development of specific antiviral [10] therapies, such as pleconaril and pocapavir, holds potential for improving outcomes in severe cases [1]. In conclusion, a multidisciplinary approach, combining early diagnostics, effective management strategies, and long-term follow-up, is essential to optimize outcomes for neonates affected by EV infections [1].

Our study has the obvious limitation of a very small case series, which necessarily means that the data cannot be generalized [6]. However, as emphasized by WHO, it is a priority [5] to report individual experiences so that we have as much data as possible to guide clinical practice and lay the foundation for larger clinical trials. Additional limitations include the retrospective, two-center design without a denominator population, which precludes estimation of incidence or diagnostic yield; the absence of maternal or contact virology, serotyping, or sequencing, and of a formal epidemiological investigation (contact tracing, cohorting, or outbreak assessment), which limited our ability to characterize the twin cluster (Patients 8–9) beyond descriptive reporting; the attribution of neuroimaging findings in the preterm twins to EV infection alone, when prematurity, growth restriction, and perinatal instability are plausible contributing factors; heterogeneous IVIG dosing across centers without a documented within-center clinical rationale; incomplete and non-standardized long-term follow-up; and the inclusion of highly granular patient-level data (exact birth weight, gestational age, and a narrow admission window across only two centers) that, despite full anonymization, could theoretically permit indirect identification by treating clinicians. This was considered acceptable given informed parental consent but is noted for transparency.

## 6. Conclusions

NEVIs represent a complex clinical challenge due to their wide spectrum of manifestations. Advanced molecular diagnostics, particularly RT-PCR, have greatly enhanced the timely and accurate detection of EV infections; underreporting has been noted as a broader concern in the literature, underscoring the potential value of standardized diagnostic protocols. IVIG is commonly administered as an adjunctive treatment in managing severe cases, particularly myocarditis, although its efficacy has not been established in controlled trials and further research is needed, together with exploration of potential antiviral agents like pleconaril and pocapavir.

While diagnostic and therapeutic advancements have improved outcomes, severe NEVIs remain associated with significant morbidity and mortality. Long-term follow-up is critical for identifying and managing potential neurodevelopmental and cardiac sequelae. A multidisciplinary approach integrating early diagnosis, targeted management, and preventive strategies is essential in mitigating the burden of NEVI and improving outcomes in this high-risk population.

## Figures and Tables

**Table 1:** Clinical data of infants with NEVI.

Patient	1	2	3	4	5	6	7	8	9
**Obstetric History and** **Neonatal Characteristics**									
**Maternal/Family History ** **before delivery**	Intrapartum maternal fever	Maternal and family GI symptoms	Asymptomatic	Asymptomatic	Asymptomatic	Older brother with fever and viral rash	Asymptomatic	Monochorionic diamniotic twin pregnancy; selective intrauterine growth restriction	Monochorionic diamniotic twin pregnancy; selective intrauterine growth restriction of co-twin
**Delivery Mode**	Vaginal	Vaginal	Vaginal	C-section	Vaginal	Vaginal	C-section	C-section	C-section
**Gestational Age (weeks)**	39^+1^	37^+6^	41^+0^	38^+0^	40^+3^	40^+2^	38^+1^	32^+0^	32^+0^
**Birth Weight (grams)**	3540	3655	3835	2290	3960	2550	3450	1160	1360
**Neonatal Clinical Presentation**									
**Symptom Onset (DOL)**	7	2	5	13	5	9	30	34	34
**Symptoms**	Fever, Irritability, Poor feeding	Hypo-reactivity, Apnea, Pallor, Capillary refill >2”	Fever, Hypo-reactivity, Poor feeding, Seizures	Fever, Irritability	Fever, Hypo-reactivity, Poor feeding	Fever	Fever, Irritability, Diarrhea	Worsening respiratory dynamics, apnea, bradycardia, cutaneous mottling	Worsening respiratory dynamics, apnea, bradycardia, cutaneous mottling
**CNS Syndrome**	Meningitis	Meningitis	Meningitis	Meningitis	Meningitis	Meningitis	Meningitis	Meningoencephalitis	Meningoencephalitis
**Rash**	No	No	No	No	No	No	No	No	No
**Myocarditis**	No	Yes	No	No	No	No	No	No	No
**Hepatitis**	No	No	No	No	No	No	No	No	No
**Diagnostic workup**									
**Molecular test on CSF**	Positive	Positive	Positive	Positive	Positive	Positive	Positive	Positive	Positive
**CSF Glucose (mg/dL)**	59	63	55	59	54	62	40	56	-
**CSF Protein (mg/dL)**	73	89	55	161	177	83	82	139	-
**CSF Cell Count (cells/mm^3^)**	8	16	3	19	57	22	221	260	-
**Max CRP Value (mg/L)**	16	19	69	5.3	11.8	5.9	4.1	120	140
**WBC (×10^9^/L)**	13.54	3.58	13.97	6.17	5.83	6.98	11.76	4.52	4.98
**Platelets (×10^9^/L)**	184	260	120	347	282	357	253	321	196
**Blood/CSF Cultures**	No growth	No growth	No growth	No growth	No growth	No growth	No growth	No growth	No growth
**Cerebral Ultrasound**	Normal	Normal	Normal	Normal	Normal	Periventricular hyperechogenicity	Normal	Persistent bilateral periventricular hyperechogenicity	Persistent bilateral periventricular hyperechogenicity
**Cerebral MRI**	Normal	Normal	Normal	Normal	Normal	Normal	Normal	Diffuse deep WM T2 hyperintensity, splenium cytotoxic edema, no hemosiderin	Periventricular WM signal changes, deep medullary vein congestion, no hemosiderin
**Echocardiogram**	Normal	Dyskinesia, pericardial effusion	Normal	Minimal pericardial detachment	Minimal pericardial detachment	Normal	Minimal pericardial detachment	Patent foramen ovale without hemodynamic significance	Patent foramen ovale without hemodynamic significance
**Electrocardiogram**	Normal	Bradycardia	Extrasystoles	Normal	Bradycardia	Normal	Normal	Normal	Normal
**Max proBNP (pg/mL)**	-	3110	-	-	-	-	-	-	-
**Treatment**									
**Antibiotics**	Yes	Yes	Yes	Yes	Yes	Yes	Yes	Yes	Yes
**Antiviral Therapy**	No	No	No	No	No	No	No	No	No
**Ventilation/Oxygen**	No	No	No	No	No	No	No	No	No
**Hydrocortisone**	No	No	No	No	No	No	No	No	No
**Dobutamine**	No	Yes	No	No	No	No	No	No	No
**Phenobarbital**	No	No	Yes	No	No	No	No	No	Yes
**IVIG (g/kg)**	0.5 for 4 days	0.5 for 4 days	0.5 for 4 days	0.25 for 3 days	0.25 for 3 days	0.25 for 3 days	0.25 for 3 days	0.5 for 4 days	0.5 for 4 days

C-Section: Cesarean section; CRP: C-reactive protein; CSF: cerebrospinal fluid; DOL: day of life; EV: enterovirus; GI: gastrointestinal; IVIG: intravenous immunoglobulin; MRI: magnetic resonance imaging; WBC: white blood cells. “Ventilation/Oxygen” refers to respiratory support required specifically during the NEVI illness course; brief respiratory support provided at birth for prematurity-related causes (Patients 8–9) is reported separately in the case narratives and is not reflected in this row. Shaded rows indicate section headers within the table.

**Table 2:** Summary of the literature review.

Reference	Number of Infants	Obstetric History	Neonatal Characteristics	Neonatal Clinical Presentation	Diagnostic Tests	Neuroimaging	Cardiac Imaging	Treatment
Morriss et al., 2016 [7]	5 neonates	Maternal viral illness, chorioamnionitis, prepartum respiratory infection; one home birth with a sibling having recent diarrhea	Born in late summer/fall; one term, one early preterm, three late preterm (34–37 weeks); birth weights 1.7–3.7 kg	Sepsis-like presentation (100%), Hypothermia (40%), fever (20%) Periventricular leukomalacia and encephalomalacia (20%) Myocarditis (40%) No cases of hepatitis (0%)	CSF PCR positive for EV in all cases; sterile bacterial cultures; elevated CRP, transient thrombocytopenia in 2 cases	One case with PVL evolving to encephalomalacia; MRI with hemorrhagic foci in lateral ventricles in one case	Echocardiography confirmed myocarditis in 2 cases; ventricular dysfunction, pericardial effusion	IVIG (3–5 g/kg) given to 2 cases with myocarditis; supportive therapy for all; antibiotics initially used but later stopped
Ghabouli Shahroodi et al., 2016 [9]	35 neonates	Not explicitly detailed in the study	Term and preterm neonates	Enteroviral meningitis 37.1%, CSF pleocytosis 23%, PMN dominance (>50%) 50%, 12.5% mortality in neonates with very low birth weight (<1500 g), 25% had brain damage and were diagnosed with severe asphyxia, 30% had hyponatremia (potential SIADH)	CSF RT-PCR positive for enteroviruses; qualitative CRP negative in 72.7% of neonates	Not specifically detailed	Not mentioned	Supportive care; mean length of hospital stay of 4.3 days among neonates with a mild course (discharged with diagnosis of culture-negative sepsis)
Harik & DeBiasi, 2018 [1]	Multiple studies, including a cohort of 338 neonates	Maternal viral illness symptoms often present before or after delivery	Primarily full-term neonates, uncomplicated postnatal courses before disease onset	Benign disease (pneumonia, respiratory illness, febrile illness, exanthema, GI illness) 26%, aseptic meningitis 47%, severe disease 27% (among clinically apparent EV infection cases)	RT-PCR for enterovirus and parechovirus; serologic assays have limited sensitivity	Not specifically detailed	Echocardiography for suspected myocarditis	Supportive care, Investigational antiviral agents: pleconaril, pocapavir, IVIG used in some cases but not proven in large trials
Chuang & Huang, 2019 [10]	146 neonates	Maternal history of viral illness, often with fever and abdominal pain; possible vertical transmission	Prematurity, early age of onset (<7 days), male sex, absence of maternal antibodies	Aseptic meningitis 41.8% Hepatic necrosis with coagulopathy 28.7% Non-specific febrile illness 29.5%	RT-PCR, viral culture (stool, CSF, nasopharynx, blood), serology	MRI/CT findings may show periventricular leukomalacia or white matter injury	Echocardiography for detecting myocarditis, cardiomegaly, ventricular dysfunction	Supportive care, IVIG, experimental antivirals (pleconaril, pocapavir), ECMO for severe myocarditis
Olchawa-Czech et al., 2021 [11]	10 neonates	Positive family history of viral infections in most cases	Premature and term neonates	Fever 80%, viral sepsis 10%, myocarditis with arrhythmia and circulatory failure 20%, meningitis with seizures 70%, hyperesthesia, apathy, respiratory distress, abdominal distension 50%, gastrointestinal symptoms 40%	RT-PCR confirmed enterovirus in cerebrospinal fluid (3 patients) and stool (5 patients) One patient had enteroviral antibodies	Thalamostriate vasculopathy in one case, MRI: intracranial hemorrhagic lesions, white matter immaturity. Normal findings in most cases	Echocardiography: myocardial dysfunction, cardiac dilatation, pericardial effusion, elevated troponin T in myocarditis cases	Supportive care, antibiotics initially used but discontinued after negative bacterial cultures, inotropic support, antiviral therapy (aciclovir) in some cases
Zhang et al., 2021 [4]	Multiple studies, including a cohort of 237 neonates	29.5% had maternal illness before delivery, 6.8% had siblings or friends with symptoms	Severe cases more frequently had lower gestational age and earlier onset of symptoms	Hepatitis/Coagulopathy: 46.0%, myocarditis 37.1%, meningoencephalitis 11.0%, other rare complications: 5.9%	RT-PCR for enterovirus from blood, CSF, stool, and respiratory samples	Among meningoencephalitis cases: MRI white matter injury in 80.8% and periventricular echogenicity on ultrasound in 61.5%	Echocardiographic findings included ventricular dilation and function depression	Blood transfusions, IVIG, mechanical ventilation, ECMO, antivirals Pleconaril, Pocapavir
Bucci et al., 2022 [2]	30 neonates	Full-term neonates, normal delivery	Mean age: 15.8 days, 56.7% male	100% fever, irritability (36.7%), poor feeding (23.3%), diarrhea (6.7%)	RT-PCR for EV and Parechovirus, CSF analysis	MRI: abnormalities in 34.6% (dural contrast, white matter alterations)	No specific cardiac imaging data	IVIG therapy in cases of viral meningitis, symptomatic treatment
Xuan et al., 2025 [12]	193 neonates	Higher prevalence of preterm birth and perinatal infection in severe cases	Lower gestational age and earlier onset (≤7 DOL) in severe infections	Sepsis-like syndrome; severe cases complicated by myocarditis, necrotizing hepatitis, disseminated intravascular coagulation, and multiorgan dysfunction	RT-PCR for enterovirus (CSF, blood, stool)	Brain MRI performed in selected cases; long-term neurological sequelae reported among survivors of severe infection	Echocardiography showing myocardial dysfunction in severe cases	Supportive intensive care; IVIG, mechanical ventilation, vasoactive drugs, blood products; no specific antiviral therapy

CNS: central nervous system, MRI: magnetic resonance imaging, IVIG: intravenous immunoglobulin, CSF: cerebrospinal fluid. Two studies included in this table (Harik & DeBiasi, 2018 [1] ; Bucci et al., 2022 [2]) report combined enterovirus and human parechovirus populations rather than enterovirus-only data; this should be considered when interpreting the summarized findings, as our own cohort includes enterovirus-confirmed cases exclusively.

## Data Availability

The data presented in this study are available on request from the corresponding author. The data are not publicly available due to privacy restrictions related to patient confidentiality.
